# Prison Labour

**Published:** 1896-05-30

**Authors:** 


### Modern Sociology.

### PRISON LABOUR.

Observations of the Prison Commissioners on the Recommendation of the Departmental Committee on Prisons appointed by the Secretary of State on June 6, 1894.

In this paper, issued a short time since, the Prison Commissioners reply to the report issued a year ago by the Departmental Committee. They express regret that the committee have not helped them in their suggestions with regard to unproductive labour by any practical suggestions. As to the work on cranks and treadwheels, they point out that in some cases water is pumped and corn is ground by these means. Out of the total number of prisons there are thirty-six where the labour is purely mechanical and unproductive, and that it is undesirable both from the point of view of the waste of labour involved and possibly of the moral effect on the mind of a prisoner condemned to a useless task. Thirty-six certainly does seem a very large number of such prisons. They, however, consider that it is of the highest importance that penal labour of a deterrent character should accompany the earlier stages of imprisonment. They propose to retain the cranks and treadwheels which pump and grind, and to utilise the others or abolish them altogether, and substitute some other form of hard labour, such as the heavy loom or stone-breaking, &c., and to limit somewhat the period of such labour. They think the "vagabond" class should be subjected to it, as they often openly prefer the prison to the workhouse. On the subject of productive labour they are quite in accord with the general recommendations of the committee, but point out that the total area within the boundaries of local prisons is 166 acres, of which only 70 acres could possibly be used by prisoners for cultivation; 59 acres are already in actual cultivation, leaving only 11 acres in the 56 local prisons which could be so used, and they propose to instruct the governors to bring such into cultivation wherever practicable. Of the 1,018 acres of land connected with the convict prisons, 973 are actually under cultivation, and nearly the whole of the remaining 45 acres is at Parkhurst Prison, where the majority of the prisoners are physically incapable of doing labourers' work, and at most 15 acres of the land, now used as pasture, could be cultivated. At Dartmoor 959 acres are under reclamation. As regards Government departments, every effort is being made to secure additional orders. On the whole, as regards the general question of prison industry, the Commissioners believe that there is a large field for useful reform, but that the steps taken must be gradual and tentative. The Commissioners advise that steps should be taken to impress upon governors that one of their chief duties, and one upon which their fitness for promotion will be judged, will consist in an intelligent supervision of and interest in the industrial management of their establishments. They are in favour of associated labour under proper supervision, and it would be of great advantage for the purpose of concentrating labour or some special form of industry, from an industrial point of view, if it could be placed under some skilled supervision in cases where such industry is not adapted to isolated work in the cells. They call attention to the difficulties which beset associated work to be taught in classes, saying that some prisoners would regard it not at all as a "welcome relief" to be obliged to associate with other criminals. Finally, they direct attention to the great differences existing between local and convict prisons. They point out that association for productive work and technical instruction will involve the building of additional workshops, providing instructors, and increasing the number of officers for purposes of supervision. They give a return showing the number of prisoners employed on May 23rd, 1895, showing that out of 3,339 male prisoners liable to first-class labour, 640 were employed upon unproductive labour, treadwheel, and crank; and that out of 6,309 male and 2,129 female prisoners who were liable to second-class hard labour, 1,137 males and 139 females were employed at oakum picking, by reason of there being no industrial work at which they could be employed. As regards farming and land reclamation a new prison would have to be built if the recommendations of the committee are to be carried out, involving a cost of from £60,000 to £80,000, and it would also involve the appointment of a new staff from the governor downwards, thus greatly increasing the annual charge for prisons. They also point out that "land reclamation" is labour of the hardest and most penal kind now reserved for prisoners sentenced to penal servitude, and that, on the contrary, farming and gardening are labour of the lightest and most pleasant kind reserved for the best conducted prisoners or for medical cases, and that only a very small number of prisoners can be so employed. They are in favour of allowing all prisoners to earn gratuities throughout their sentences, both local and convict. They are also in favour of a system under which the special or extra gratuities now given for good conduct would be granted as a reward for specially good or extra work, the prisoner thus receiving a percentage on the value of the good or special work done by him. An extension of the present mark system would effect this. They admit the desirability of improving the rank of the official who supervises the industrial work of the prisons, in order that he may have more authority and responsibility, and suggest that he should have the title of "Comptroller of Industries," with the status and salary of an inspector of prisons. They also suggest that an extra allowance should be only given to the officers who not only qualify but are actually employed supervising an industry requiring a certain amount of skill.

Such, in brief, are the remarks of the Prison Commissioners on the recommendations of the Departmental Committee, as far as the question of "prison labour" is concerned. The pamphlet of 48 pages is full of interesting information on all that concerns prison life, and the Commissioners who sign it have evidently taken the greatest possible care to inquire into the feasibility of carrying out as many of the recommendations of the Departmental Committee as in the nature of the case may be found practicable.

